# The Complex Journey of Women in Perinatal Psychiatric Care: Susceptibility to Illness Onset, Comorbidity and Clinical Trajectories: Le parcours complexe des femmes en psychiatrie périnatale : vulnérabilité, comorbidités et trajectoires cliniques

**DOI:** 10.1177/07067437251328347

**Published:** 2025-04-16

**Authors:** Alexandra Painchaud, Marie-Josée Poulin, Célia Matte-Gagné, Chantal Mérette

**Affiliations:** 1 Centre de recherche CERVO, Québec City, QC, Canada; 2 École de psychologie, 4440 Université Laval, Québec City, QC, Canada; 3 177450 Institut universitaire en santé mentale de Québec, Québec City, QC, Canada; 4 Département de psychiatrie et neurosciences, 4440 Université Laval, Québec City, QC, Canada; 5 Groupe de recherche sur l’inadaptation psychosociale chez l’enfant (GRIP), Quebec City, QC, Canada; 6 Centre de recherche universitaire sur les jeunes et les familles (CRUJeF), Quebec City, QC, Canada; 7 Centre de recherche du CHU de Québec, 4440 Université Laval, Québec City, QC, Canada

**Keywords:** perinatal psychiatric disorders, pregnancy, postpartum, incidence, comorbidity, clinical trajectories

## Abstract

**Background:**

More than one in five women deal with a psychiatric disorder during the perinatal period. Whereas perinatal depression is well documented, there is still little research on the full range of perinatal psychiatric disorders and their clinical evolution across this whole period. The present study investigated the susceptibility to psychiatric illness during pregnancy and up to one year postpartum. We aimed to identify the most frequent disorders and comorbidities arising in each perinatal period. We outlined the clinical trajectories of these disorders in terms of evolution across past history, pregnancy and postpartum.

**Method:**

Through a retrospective longitudinal design, data were collected in 2019–2020 from the medical records of the cohort of 964 women who required care in a tertiary perinatal psychiatry clinic located in Quebec City (Canada) between 2004 and 2020. Incidence rates of the full range of psychiatric disorders were estimated per period and their evolution across time identified clinical trajectories.

**Results:**

During pregnancy, 34 different disorders were newly diagnosed with incidence rates ranging from 0.1% to 15.5% (45.6% of women having had at least one disorder diagnosed during pregnancy) whereas, during postpartum, 36 disorders were newly diagnosed with incidence rates ranging from 0.1% to 31.0% (67.5% of women having had at least one disorder diagnosed during postpartum). For most disorders, rates were significantly higher in postpartum than in pregnancy. A woman could develop multiple disorders during a given perinatal period: this comorbidity involved various combinations of diagnoses in 28% of women during pregnancy and 38% during postpartum. We outlined 52 different clinical trajectories from past history to postpartum, underlining the heterogeneity of the perinatal course.

**Conclusions:**

Pregnancy is a susceptible period for women with past psychiatric histories whereas postpartum could trigger a new illness in women without a past history or pregnancy-onset psychiatric disorder.

## Résumé

**Contexte:**

Plus d’une femme sur cinq souffrirait d’un trouble psychiatrique au cours de la période périnatale. Alors que la dépression périnatale est bien documentée, il y a encore peu de recherche sur la gamme complète de troubles psychiatriques périnataux et leur évolution clinique sur l’ensemble de cette période. La présente étude vise à explorer la susceptibilité aux troubles psychiatriques pendant la grossesse et la première année postnatale. L’objectif était d’identifier les troubles et comorbidités les plus fréquents à chaque période périnatale. Nous avons décrit les trajectoires cliniques de ces troubles en termes d’évolution à travers les antécédents, la grossesse et le post-partum.

**Méthodologie:**

Dans le cadre de cette étude longitudinale rétrospective, les données ont été recueillies en 2019–2020 à partir des dossiers médicaux de la cohorte de 964 femmes ayant nécessité des soins dans une clinique spécialisée de psychiatrie périnatale de la ville de Québec entre 2004 et 2020. Les taux d’incidence de l’ensemble des troubles psychiatriques ont été estimés pour chaque période, et leur évolution au fil du temps a permis d’identifier les trajectoires cliniques.

**Résultats:**

Pendant la grossesse, 34 troubles ont été nouvellement diagnostiqués avec une incidence allant de 0,1 à 15,5% (45,6% des femmes ayant eu au moins un trouble diagnostiqué pendant la grossesse), et pendant la période postpartum, 36 troubles ont été nouvellement diagnostiqués avec une incidence variant de 0,1 à 31,0% (67,5% des femmes ayant connu au moins un trouble diagnostiqué en postpartum). Pour la plupart des troubles, les taux étaient significativement plus élevés pendant le postpartum que la grossesse. Une femme pouvait développer plusieurs troubles au cours d’une même période périnatale : cette comorbidité concernait diverses combinaisons de diagnostics chez 28% des femmes pendant la grossesse et 38% en post-partum. Nous avons identifié 52 trajectoires cliniques distinctes, des antécédents au post-partum, soulignant ainsi l’hétérogénéité du parcours périnatal.

**Conclusions:**

La grossesse serait une période à risque pour les femmes ayant des antécédents psychiatriques, tandis que le post-partum pourrait déclencher un nouveau trouble chez celles sans antécédents ni incidence pendant la grossesse.

## Introduction

Perinatal psychiatry deals with the management of mental health disorders in women during pregnancy and the postpartum period. During these periods, the risk of developing a psychiatric disorder could increase, affecting more than one in five women, especially for women with a preexisting past psychiatric history.^1–6^ For instance, pregnancy could be a period of vulnerability for the onset of mood disorders with the estimated incidence of prenatal depression reaching 20.7% according to systematic reviews and meta-analyses.^7,8^ However, this rate varies across studies due in part to the difficulty of diagnosing a psychiatric disorder when women are only screened once throughout pregnancy.^9^ Assessing mental health at multiple points such as prior to conception, during pregnancy and postpartum periods, may enhance our understanding about the onset and course of perinatal disorders.

When the postpartum period is defined as the first year after childbirth, the risk of mood disorders was also found increased in several studies. For instance, a large meta-analysis involving 1,236,365 women from 80 countries reported that postpartum depression was found in 17.2% of the world's population.^10^ Moreover, women are 1.59 times more likely to develop depression during postpartum than childbearing age nulliparous women,^11^ and 1.15 times more likely than pregnant women.^12^ Noteworthy, more than 80% of the studies on postpartum depression used the Edinburgh Postnatal Depression Scale (EPDS) as their diagnostic tool,^10^ relying on self-reported retrospective recall of symptomatology, rather than clinical diagnoses, which may misestimate prevalence^13^ and omit the assessment of comorbid diagnoses.^14^

Whereas postpartum depression has already been well documented, other diagnoses can also appear during the perinatal period, such as bipolar and anxiety,^3,5,14,15^ obsessive-compulsive,^16,17^ substance use,^18,19^ personality,^20,21^ or psychotic^11,12,22^ disorders. However, previous studies have either focused on the pregnancy or postpartum period, or maybe both, but mostly in a cross-sectional design. Some have followed women across the perinatal period and found rising rates of diagnosed psychiatric disorders mainly during the postpartum.^12,22–24^ Nevertheless, as acknowledged by Howard and Khalifeh (2020), there is still little research on the full range of perinatal psychiatric disorders, and none described their clinical evolution across this whole period.

Depression and anxiety disorders are highly comorbid during pregnancy^7^ and postpartum.^4,26,27^ Nevertheless, as suggested by Agius et al. (2016), there is still a lack of research on multi-comorbidity, i.e., the coexistence of more than two perinatal psychiatric disorders. Moreover, some studies have reported that screening may be limited to depression during the perinatal period and that other comorbid disorders may then be undetected, leading to inadequate treatment and psychological and medical long-term effects for both mother and child.^14^^,29,30^

This study is specifically designed to describe a cohort of women who required care in a tertiary perinatal psychiatry clinic. Our aim to quantify the incidence of newly diagnosed psychiatric disorders during pregnancy and postpartum periods will allow us to identify the most frequent disorders for each period and the most vulnerable period for each disorder. This cohort will also help describe perinatal comorbidities and outline the clinical trajectories of the full range of psychiatric disorders in terms of evolution across past history, pregnancy and postpartum periods.

## Methods

### Participants and Design

Our retrospective longitudinal descriptive cohort study included the cohort of 964 women who required specialized care in a tertiary perinatal psychiatry clinic, located in Quebec City (Canada), at some point between 2004 and 2020. The clinic offers assessment, care, and treatment services to women who are in preconception, pregnant or in their first year postpartum, and who have had a previous or an active psychiatric condition. The inclusion criterion was to have had at least one consultation during this timeframe, whether or not the follow-up covered the whole perinatal period. There were no exclusion criteria given that all women who received care at the clinic were included. According to the best practice in the field,^31,32^ data were collected in 2019–2020 from a review of the women's medical records by a psychology PhD student (AP) supervised by the psychiatrist-in-charge of the perinatal psychiatry clinic (MJP). Ethical approval was obtained from the Neuroscience and Mental Health Research Ethics Committee of the *Centre intégré univerisitaire de santé et de services sociaux de la Capitale-Nationale* (CIUSSS-CN; project # 2020–1851).

At their first consultation, 89 women from the cohort (9%) were in preconception though only 52 of them eventually got pregnant, 514 women (53%) were pregnant, and 361 women (38%) already in the postpartum period also provided medical information regarding past history and pregnancy. This yielded a sample of 927 women for studying the pregnancy period and, when including those with medical information regarding postpartum, a subsample of 772 women for studying the postpartum period.

### Measures

#### Cohort Characteristics

Sociodemographic, familial and medication information was sought from medical records and included: age at childbirth, nativity, marital status, parity and obstetric information, familial psychiatric history, and the psychotropic medication used during pregnancy and postpartum. Psychotropics belonging to benzodiazepine, non-benzodiazepine anxiolytic hypnotic and psychostimulant categories were grouped under those categorical labels. Also, medication could have been prescribed before the consultation in perinatal psychiatry and could have been discontinued or modified during the perinatal follow-up.

#### Psychiatric Diagnoses

For all women in the cohort, the medical record was used to document the past psychiatric history, whereas new psychiatric disorders diagnosed during the perinatal period were based on semi-structured interviews (modified SCID) conducted by the psychiatrist-in-charge of the clinic (MJP). Hence, these newly diagnosed disorders, that were not included in the woman's past history or otherwise that were known to be resorbed, contributed to the incidence of a psychiatric disorder with a timing of onset either during pregnancy or within one year postpartum.

Currently, there is no unique time frame for labelling a postpartum depression. The American Psychiatric Association's Diagnostic and Statistical Manual of Mental Disorders (**DSM-5**) defines “peripartum onset” as a specifier of a major depressive disorder when symptom onset occurs during pregnancy or in the four weeks following delivery, whereas the Centers for Disease Control and Prevention (**CRD**) and the American College of Obstetricians and Gynecologists (**ACOG**) extend the postpartum period to 12 months after childbirth.^6^ In our study, we distinguished a depressive disorder that began during pregnancy versus up to one year postpartum, referring to prenatal vs. postpartum depression, respectively, as recently suggested by Molenaar et al. (2023) and often referred to in research.^10^

Psychiatric disorders were not exclusive and diagnoses that have occurred concomitantly during a same perinatal period are referred to as perinatal onset comorbidities or multi-comorbidities.

### Statistics

Analyses were performed using SPSS Statistics 29.0 and SAS 9.4M8. To describe the sociodemographic, familial and medication profile of this cohort, we reported the mean (**M**), standard deviation (**SD**) and range of quantitative characteristics, or frequency and percentage of categorical ones.

The prevalence rate (%) of a past psychiatric disorder history was defined by the observed frequency of women who had been diagnosed with this disorder before pregnancy among all 964 women of the cohort. The incidence rate (%) of a psychiatric disorder during pregnancy was defined by the observed frequency of this newly diagnosed disorder divided by the sample of 927 women for whom the pregnancy period was documented, whereas the incidence rate of a psychiatric disorder during postpartum was defined by the observed frequency of this newly diagnosed disorder divided by the subsample of 772 women who were followed up to one year postpartum. Hence, in a woman, a same disorder could only be newly diagnosed during pregnancy or postpartum, and thus contribute to the incidence of only one of these periods. Noteworthy, a woman could have had multiple disorders newly diagnosed within a same period and, therefore, contribute to the incidence of more than one diagnosis. Consequently, the sum of all the percentages is not interpretable and can exceed 100%. To identify the most vulnerable perinatal period for each diagnosis, Chi-square or Fisher's exact tests were used to compare pregnancy vs. postpartum incidence rates.

Cross-tabulations of disorders within a same perinatal period were used to identify comorbidities, i.e., the most frequent combinations of disorders diagnosed within a same woman. For this exercise, all anxiety disorders were grouped together, as well as all personality and psychotic disorders (see Table 3 for diagnostic groupings). An anxiety or personality disorder involved in a comorbidity meant that a woman had at least one disorder within these diagnostic families and may have had several diagnoses within a same family (e.g., panic disorder and agoraphobia). Likewise, to outline clinical trajectories, cross-tabulations of all diagnosis combinations across time were carried out and weighted according to the number of women following a given trajectory. Notably, the trajectories were not exclusive since a woman may have expressed more than one disorder during each period.

## Results

### Cohort Characteristics

The characteristics of the entire cohort of 964 patients are shown in Table 1. At childbirth, the women were aged between 17 and 44 (M = 30.5 years, SD = 4.9). Most of them were born in Canada (90.5%), were in a common-law relationship (71.4%, a cultural particularity of Quebec, Canada), and had a family psychiatric history (in at least one first-, second-, or third-degree family member; 74.3%). Also, 23.0% of pregnancies were unplanned and 3.0% were considered undesired.

**Table 1.** Characteristics of a Cohort of Women Who Required Psychiatric Care in a Tertiary Perinatal Psychiatry Clinic (N = 964).

Characteristics	M (SD) or n (%)	Range
**Age at childbirth**	30.5 (4.9)	17–44

**Nativity**		
Canada	872 (90.5)	
North America (excluding Canada)	4 (0.4)	
South or Central America	24 (2.5)	
Europe	32 (3.3)	
Africa	22 (2.3)	
Asia	4 (0.4)	
*Unknown*	6 (0.6)	

**Marital status**		
Common-law partner	688 (71.4)	
Married	152 (15.8)	
Single	53 (5.5)	
Separated or divorced	46 (4.8)	
Widow	5 (0.5)	
*Unknown*	20 (2.1)	

**Obstetric state at first consultation**		
Preconception psychiatric consultation	89 (9.2)	
Pregnancy	514 (53.3)	
First trimester	56 (10.89)	
Second trimester	213 (41.44)	
Third trimester	220 (42.80)	
*Unknown*	25 (4.86)	
Postpartum	361 (37.5)	
0–6 weeks	82 (22.71)	
2 months	39 (10.80)	
3 months	51 (14.13)	
4 months	37 (10.25)	
5 months	24 (6.65)	
6 months	23 (6.37)	
7 months	29 (8.03)	
8 months	12 (3.32)	
9 months	10 (2.77)	
10 months	11 (3.05)	
11 months	3 (0.83)	
12 months	23 (6.37)	
*Unknown*	17 (4.71)	

**Parity**		
Primiparous	520 (53.9)	
Multiparous	376 (39.0)	
Second child	269 (71.54)	
Third	79 (21.01)	
Fourth or more	28 (7.45)	
* No pregnancy or interrupted pregnancy*	50 (5.2)	
* Unknown*	18 (1.9)	

**Pregnancy**		
Planned	279 (29.0)	
Unplanned	222 (23.0)	
*Unknown (or not applicable)*	463 (48.0)	

Wanted		
Both parents	522 (54.0)	
Mother only	55 (6.0)	
Father only	15 (2.0)	
Unwanted	28 (3.0)	
*Unknown (or not applicable)*	344 (36.0)	

**Familial psychiatric history**		
Yes	716 (74.3)	
No	81 (8.4)	
*Unknown*	167 (17.3)	

At their first consultation, slightly more than half of patients were primiparous (53.9%). Among multiparous women (n = 376), 14.0% had experienced a psychiatric episode during a previous pregnancy, 35.0% during a previous postpartum period, and 9.0% during both previous perinatal periods; these episodes had been treated in another clinic.

Among the cohort of women, 353 were under pharmacological treatment during pregnancy and 613 during postpartum. The distribution of the psychotropic medication used is presented in Table 2 and shows that, for both the pregnancy and postpartum period, Citalopram, Benzodiazepines (mainly Lorazepam, Temazepam and Oxazepam), Sertraline and Quetiapine, were the most frequently used, non-exclusively, ranging from 18.7 to 26.9%.

**Table 2.** Distribution of the Type of Psychotropic Medication Used^1^ Among Women Under Pharmacological Treatment During Pregnancy (n = 353) and Postpartum Period (n = 613).

**Psychotropic medication**	**Pregnancy ** (Subgroup of N = 353 women using medication during pregnancy)	**Postpartum ** (Subgroup of N = 613 women using medication during postpartum)
	**n (%)**	**n (%)**
**Antidepressants – Selective Serotonin Reuptake Inhibitors (SSRIs)**		
Sertraline	75 (21.3)	142 (23.2)
Citalopram	82 (23.2)	163 (26.6)
Escitalopram	41 (11.6)	90 (14.7)
Fluoxetine	7 (1.2)	46 (7.5)
Fluvoxamine	4 (1.1)	9 (1.5)
Paroxetine	23 (6.5)	39 (6.4)
**Antidepressants – Serotonin-Norepinephrine Reuptake Inhibitors (SNRIs)**		
Venlafaxine	50 (14.2)	108 (17.6)
Desvenlafaxine	4 (1.1)	23 (3.8)
Levomilnacipran	0 (0.0)	2 (0.3)
Duloxetine	1 (0.3)	3 (0.5)
**Other antidepressants**		
Mirtazapine	2 (0.6)	3 (0.5)
Buproprion	16 (4.5)	62 (10.1)
Desipramine	1 (0.3)	1 (0.2)
Clomipramine	1 (0.3)	2 (0.3)
Amitriptyline	5 (1.4)	4 (0.7)
Nefazodone	0 (0.0)	1 (0.2)
Nortriptyline	2 (0.6)	2 (0.3)
Vortioxetine	0 (0.0)	7 (1.1)
**Mood stabilizers**		
Lithium	5 (1.4)	63 (10.3)
Epival	3 (0.9)	8 (1.3)
Carbamazepine	0 (0.0)	1 (0.2)
Lamotrigine	11 (3.1)	16 (2.6)
**First-generation antipsychotics**		
Haloperidol	3 (0.9)	3 (0.5)
Perphenazine	1 (0.3)	2 (0.3)
Trifluoperazine	1 (0.3)	0 (0.0)
**Second- and third-generation antipsychotics**		
Aripiprazole	10 (2.8)	28 (4.6)
Brexpiprazole	1 (0.3)	1 (0.2)
Asenapine	1 (0.3)	1 (0.2)
Lurasidone	1 (0.3)	17 (2.8)
Ziprasidone	0 (0.0)	1 (0.2)
Risperidone	3 (0.9)	17 (2.8)
Olanzapine	50 (14.2)	110 (17.9)
Clozapine	0 (0.0)	1 (0.2)
Quetiapine	66 (18.7)	122 (19.9)
**Anticonvulsants**		
Gabapentine	0 (0.0)	1 (0.2)
**Antiparkinsonians**		
Procyclidine	0 (0.0)	9 (1.5)
**Anxiolytics, sedatives and hypnotics**		
Benzodiazepines^2^	80 (22.7)	165 (26.9)
Trazodone	6 (1.7)	44 (7.2)
Non-benzodiazepine anxiolytic hypnotics^2^	4 (1.1)	17 (2.8)
**Psychostimulants** ^2^	3 (0.9)	26 (4.2)
**Non-stimulants**		
Atomoxetine	0 (0.0)	1 (0.2)

^1^ It should be noted that medication could have been prescribed before the consultation in perinatal psychiatry and could have been discontinued or modified during the perinatal follow-up.

^2^ Psychotropics belonging to benzodiazepine, non-benzodiazepine anxiolytic hypnotic, and psychostimulant categories were grouped under those categorical labels.

### Incidence of Perinatal Psychiatric Disorders

The incidence rates of psychiatric disorders newly diagnosed during pregnancy and postpartum are detailed in Table 3. During pregnancy, prenatal depression (15.5%) and generalized anxiety disorder (12.6%) were the most frequent disorders newly diagnosed, whereas during the postpartum period, it was postpartum depression (31.0%), bipolar disorder (12.2%), and generalized anxiety disorder (11.0%). Several other disorders were observed with a rate of less than 10% and ranged from substance-induced psychotic disorder (0.1%) to obsessive-compulsive disorder (9.5%).

**Table 3.** Prevalence of Psychiatric Disorders Diagnosed Before Pregnancy (Past History) and Incidence of Disorders Newly Diagnosed^1^ During Pregnancy and Postpartum Periods Among Women of the Whole Cohort (N = 964), Those for Whom the Pregnancy Period was Documented (n = 927), and Those Followed up to One Year Postpartum (n = 772), Respectively.

	Before pregnancy (Past history) (N = 964)		Pregnancy (n = 927)	Postpartum (n = 772)	*P*-value^3^ from the comparison of diagnoses according to timing of onset (pregnancy vs. postpartum)
Diagnosis	n (%)^2^		n (%)	n (%)	
***Depressive disorders***	357 (37.0)		**234** (**25.2)**	**332** (**43.0)**	**< 0.001**
Prenatal depression^4^	9 (0.9)		144 (15.5)	N/A	N/A
Postpartum depression^4^	99 (10.3)		N/A	239 (31.0)	N/A
Major depressive disorder	235 (24.4)		28 (3.0)	24 (3.1)	0.99
Persistent depressive disorder	31 (3.2)		42 (4.5)	40 (5.2)	0.82
Premenstrual dysphoric disorder	23 (2.4)		**24** (**2.6)**	** 57** (**7.4)**	**< 0.001**
Psychotic depression	11 (1.1)		1 (0.1)	6 (0.8)	0.05
Seasonal affective disorder	9 (0.9)		7 (0.8)	7 (0.9)	0.94

***Bipolar disorders***	112 (11.6)		** 47** (**5.1)**	** 94** (**12.2)**	**< 0.001**
Type I	52 (5.4)		**12** (**1.3)**	** 35** (**4.5)**	**< 0.001**
Type II	41 (4.3)		**26** (**2.8)**	** 47** (**6.1)**	**< 0.001**
Unspecified	21 (2.2)		8 (0.9)	16 (2.1)	0.11
** **Cyclothymic disorder	4 (0.4)		1 (0.1)	1 (0.1)	1.00

***Psychotic disorders***	40 (4.1)		** 7** (**0.8)**	** 24** (**3.1)**	**< 0.001**
Postpartum psychosis^4^	6 (0.6)		N/A	15 (1.9)	N/A
Schizophrenia	11 (1.1)		4 (0.4)	3 (0.4)	1.00
Schizoaffective disorders	14 (1.5)		3 (0.3)	6 (0.8)	0.31
Substance-induced psychotic disorder	15 (1.6)		0 (0.0)	1 (0.1)	0.45

***Anxiety disorders***	219 (22.7)		218 (23.5)	175 (22.7)	0.92
Generalized anxiety disorder	99 (10.3)		117 (12.6)	85 (11.0)	0.59
Panic disorder	55 (5.7)		**58** (**6.3)**	**25** (**3.2)**	**0**.**02**
Agoraphobia	37 (3.8)		59 (6.4)	48 (6.2)	0.99
Social phobia	26 (2.7)		57 (6.1)	42 (5.4)	0.82
Specific phobia	26 (2.7)		40 (4.3)	21 (2.7)	0.21
Unspecified anxiety disorder	69 (7.2)		37 (4.0)	45 (5.8)	0.21

***Obsessive-compulsive disorder (OCD)***	63 (6.5)		68 (7.3)	73 (9.5)	0.29
** **					
***Trauma- and stressor-related disorders***					
** **Post-traumatic stress disorder (PTSD)	49 (5.1)		32 (3.5)	32 (4.1)	0.78
Adjustment disorders	56 (5.8)		** 35** (**3.8)**	** 57** (**7.4)**	**< 0.001**

***Personality disorders***	78 (8.1)		** 65** (**7.0)**	** 91** (**11.8)**	**< 0.001**
*Cluster A*					
Schizoid personality disorder	1 (0.1)		0 (0.0)	0 (0.0)	1.00
*Cluster B*					
Antisocial personality disorder	1 (0.1)		0 (0.0)	2 (0.3)	0.21
Borderline personality disorder	63 (6.5)		** 51** (**5.5)**	**67** (**8.7)**	**0**.**04**
Histrionic personality disorder	2 (0.2)		2 (0.2)	4 (0.5)	0.42
Narcissistic personality disorder	2 (0.2)		3 (0.3)	1 (0.1)	0.63
*Cluster C*					
Avoidant personality disorder	2 (0.2)		1 (0.1)	6 (0.8)	0.05
Dependent personality disorder	6 (0.6)		2 (0.2)	3 (0.4)	0.66
Obsessive-compulsive personality disorder	5 (0.5)		7 (0.8)	14 (1.8)	0.14

Mixed personality disorder	3 (0.3)		2 (0.2)	1 (0.1)	1.00

***Attention-deficit/hyperactivity disorder***	81 (8.4)		46 (5.0)	60 (7.8)	0.06

***Somatic related disorders***	9 (0.9)		14 (1.5)	13 (1.7)	0.96

***Eating disorders***	80 (8.3)		6 (0.6)	7 (0.9)	0.83

***Sleeping disorders***	7 (0.7)		3 (0.3)	3 (0.4)	1.00

***Addictive disorders (substance and/or alcohol-related)***	160 (16.6)		4 (0.4)	8 (1.0)	0.33

**Any diagnosis**	707 (73.3)		**423** (**45.6)**	**521** (**67.5)**	**< 0.001**
No new diagnosis	91 (9.4)		** 471** (**50.8)**	**232** (**30.1)**	**< 0.001**

***Unknown***	166 (17.2)		33 (3.6)	19 (2.5)	n/A

^1^ The psychiatric disorders newly diagnosed during the perinatal period refer to disorders that were not previously reported in the woman's past history, or a new psychiatric episode when a previous one was completely resolved before the perinatal period. Therefore, a same woman can be found in only one column of a same diagnosis (past history, pregnancy or postpartum onset), except for resolvable disorders such as depression.

^2^ A woman can contribute to the prevalence of more than one diagnosis. Hence, each diagnosis is treated as a dichotomous variable (presence vs. absence) and consequently, the sum of the percentages is not interpretable and can exceed 100%.

^3^ A chi-square test was performed to compare the incidence of a new psychiatric diagnosis during pregnancy vs. during postpartum. Fisher's exact test was rather performed when an expected frequency was <5.

^4^ These diagnoses apply only to the 376 multiparous women who have already had another child before this follow-up at the tertiary perinatal psychiatry clinic.

Table 3 also provides the p-values from the comparison of each diagnosis according to their timing of onset (pregnancy vs. postpartum) and revealed that the incidence rate of panic disorder was higher during pregnancy than postpartum (*p *= 0.002), whereas the rates of depressive, adjustment, bipolar, psychotic and borderline personality disorders were significantly higher during postpartum than pregnancy (*p* ranging from < 0.001 to 0.04). The rate of being diagnosed with at least one new psychiatric disorder was significantly higher in the postpartum (67.5%) than in the pregnancy (45.6%; *p *< 0.001).

### Perinatal Onset Comorbidities

Among the cohort, 28% of women during pregnancy and 38% during postpartum presented perinatal onset comorbidities. Comorbidity arises from various combinations of disorders; those found in at least 10 women are presented in Table 4a-b. The combination of depression and anxiety disorder stood out, both during pregnancy (6.2%) and postpartum (10.1%).

**Table 4.** Comorbidity and Multi-Comorbidity (n ≥ 10) of Psychiatric Disorders Newly Diagnosed During the Pregnancy (a; n = 927) or the Postpartum (b; n = 772) Periods.

a) Pregnancy			
Comorbidity involving two diagnoses			
**First disorder**	**Second disorder**		**n** (%)
Anxiety disorder +	Prenatal depression		57 (6.2)
	Obsessive-compulsive disorder		46 (5.0)
	Personality disorder		33 (3.6)
	Attention-deficit/hyperactivity disorder		30 (3.24)
	Post-traumatic stress disorder		22 (2.4)
	Persistent depressive disorder		20 (2.2)
	Bipolar disorder		19 (2.1)

Prenatal depression +	Obsessive-compulsive disorder		19 (2.1)
	Personality disorder		17 (1.8)

Attention-deficit/hyperactivity disorder +	Personality disorder		12 (1.3)
	Obsessive-compulsive disorder		11 (1.2)
	Persistent depressive disorder		10 (1.1)
Multi-comorbidity involving more than two diagnoses			
**First disorder**	**Second disorder**	**Third disorder**	**n** (%)
Anxiety disorder +	Prenatal depression +	Obsessive-compulsive disorder	10 (1.1)
b) Postpartum			
Comorbidity involving two diagnoses			
**First disorder**	**Second disorder**		**n** (%)
Anxiety disorder +	Postpartum depression		78 (10.1)
	Obsessive-compulsive disorder		47 (6.1)
	Personality disorder		40 (5.2)
	Attention-deficit/hyperactivity disorder		30 (3.9)
	Bipolar disorder		24 (3.1)
	Adjustment disorder		19 (2.5)

Postpartum depression +	Personality disorder		36 (4.7)
	Obsessive-compulsive disorder		32 (4.2)
	Attention-deficit/hyperactivity disorder		24 (3.1)

Personality disorder +	Obsessive-compulsive disorder		20 (2.6)
	Bipolar disorder		17 (2.2)
	Attention-deficit/hyperactivity disorder		14 (1.8)

Obsessive-compulsive disorder +	Attention-deficit/hyperactivity disorder		14 (1.8)
	Bipolar disorder		10 (1.3)
Multi-comorbidity involving more than two diagnoses			
**First disorder**	**Second disorder**	**Third disorder**	
Anxiety disorder +	Postpartum depression +	Obsessive-compulsive disorder	19 (2.5)
		Personality disorder	18 (2.3)
		Attention-deficit/hyperactivity disorder	16 (2.1)

Anxiety disorder +	Obsessive-compulsive disorder +	Personality disorder	12 (1.6)
		Attention-deficit/hyperactivity disorder	11 (1.4)

Postpartum depression +	Obsessive-compulsive disorder +	Personality disorder	12 (1.6)

### Clinical Trajectories

**Figure 1.**
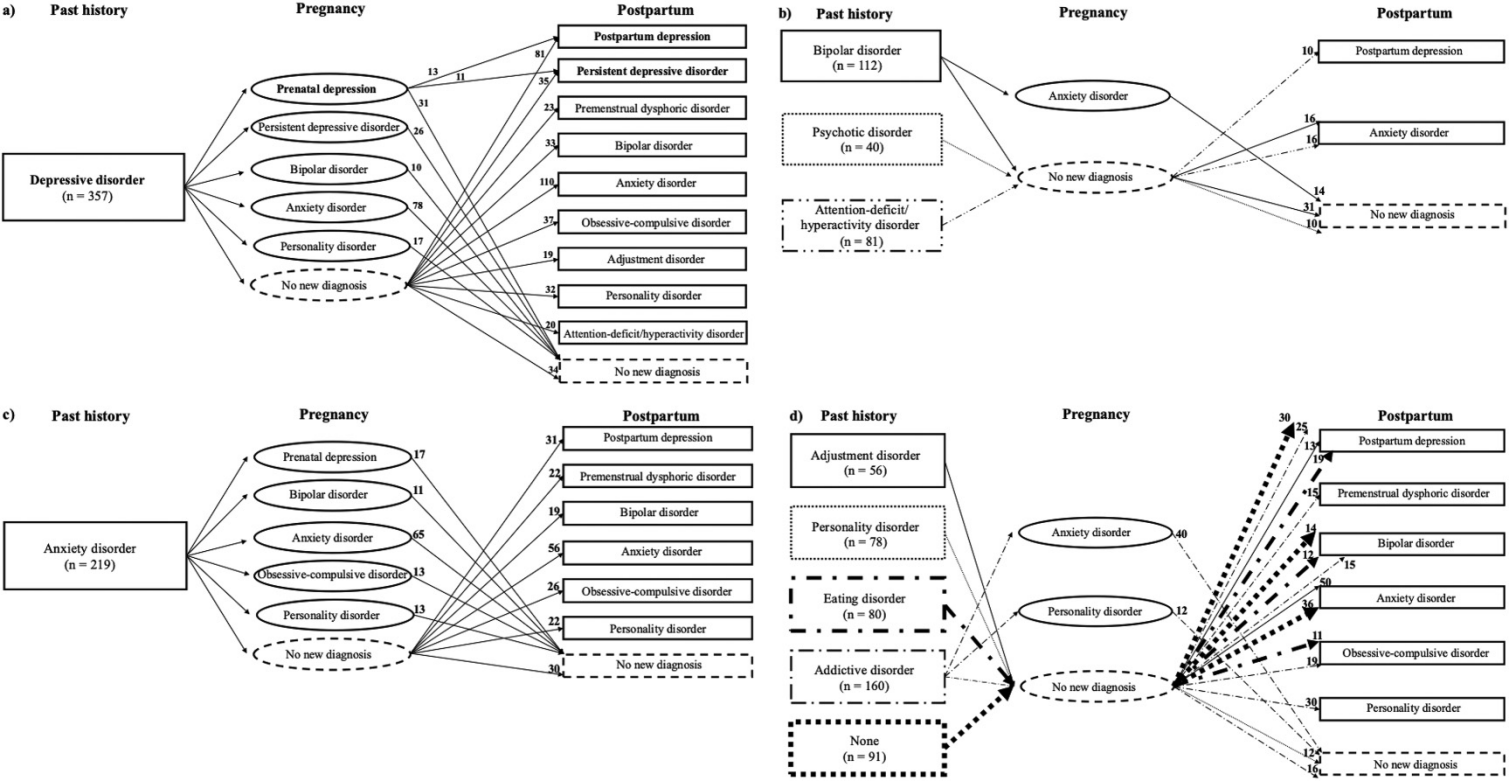
Clinical trajectories (n ≥ 10) of the evolution of newly diagnosed psychiatric disorders across past history, pregnancy and postpartum periods among the 772 women having had a medical follow-up until the postpartum, beginning with women having a past history of : a) depressive disorder, b) bipolar, psychotic or attention-deficit/hyperactivity disorders, c) anxiety disorder, and d) adjustment, personality, eating or addictive disorders, or without a past psychiatric history.

Figure 1a to 1d outline the 52 different clinical trajectories followed by a minimum of ten women, distributed according to their past psychiatric history. Most trajectories involved a disorder newly diagnosed either during pregnancy or postpartum, but not both, except for the trajectories beginning with a past depressive disorder history (see Figure 1a), which was followed by a newly diagnosed prenatal depression, and postpartum depression (n = 13) or persistent depressive disorder (n = 11), representing 3.1% of the subsample of 772 women followed until the postpartum period.

Among the trajectories involving a past psychiatric history, the following stood out: a past depressive disorder history (Figure 1a) followed by the onset of an anxiety disorder during pregnancy (n = 78; 10.1%) or postpartum (n = 110; 14.3%), or followed by the onset of a prenatal depression (n = 55; 7.1%) or postpartum depression (n = 81; 10.5%); a past bipolar disorder history (Figure 1b) followed by an anxiety disorder during pregnancy (n = 14; 1.8%) or postpartum (n = 16; 2.1%); a past anxiety disorder history (Figure 1c) followed by the onset of another anxiety disorder during pregnancy (n = 65; 8.4%) or postpartum (n = 56, 7.3%).

In a few cases, the past psychiatric history was rather followed by the absence of any new perinatal onset diagnosis: i.e., a past depressive disorder (n = 34; 4.4%), bipolar disorder (n = 31; 4.0%), anxiety disorder (n = 30; 3.9%), addictive disorder (n = 16; 2.1%), personality disorder (n = 12; 1.6%) or psychotic disorder (n = 10; 1.3%) history.

Moreover, some women with a postpartum depression (n = 30; 3.9%), a bipolar disorder (n = 14; 1.8%) or an anxiety disorder (n = 36; 4.7%) diagnosed during postpartum had no previous past psychiatric history or pregnancy-onset disorder (see Figure 1d). These were the only women in the cohort to have had a first-lifetime onset of a psychiatric disorder during the perinatal period.

## Discussion

Our study of a cohort of women who required care in a tertiary perinatal psychiatry clinic examined the full range of psychiatric disorders newly diagnosed during pregnancy or postpartum, including their comorbidities and evolution across past history and throughout the whole perinatal period, and hence expanded the general pool of knowledge over previous studies that mostly focused on depression or on patients with a perinatal relapse of another preexisting disorder.

We reported the incidence rates, of the full range of psychiatric disorders arising during the perinatal period, separately during pregnancy and postpartum. Although only two disorders had an incidence rate of at least 10% during pregnancy and three during postpartum, various psychiatric disorders showed non-exclusive incidence rates below 10%, revealing a wide range of new diagnoses during the perinatal period. Moreover, rates were mostly higher in the postpartum than during pregnancy, except for panic disorder. Similar studies reported a trend of higher rates of anxiety disorders in pregnancy versus postpartum.^5^ During pregnancy, significant hormonal fluctuations occur and can influence the balance of neurotransmitters, such as serotonin and GABA, which are involved in the regulation of anxiety.^34^ Moreover, pregnancy could particularly generate anxiety related to uncertainty and anticipatory worries about the baby's health, childbirth and ability to become a parent. On the other hand, other studies found increased psychiatric hospital admissions, perinatal relapses of a preexisting mood disorder, or incidence rates of mood and psychotic disorders during postpartum compared to pregnancy.^12,22,24^ Moreover, Mota et al. (2019)^12^ also found a higher rate of psychotic disorders during postpartum compared to the period of 40 weeks preceding pregnancy. In our study, rates of most disorders tended to be rather higher before pregnancy, but this is mainly because the past history period covered the whole lifetime and therefore was not comparable to the perinatal period. Noteworthy, nearly three-quarters of the cohort had a past history of at least one psychiatric disorder listed in Table 3, whereas nearly half and two-thirds developed a new one during pregnancy and postpartum, respectively, suggesting that entering the perinatal period with a past psychiatric history does not necessarily predispose to new issues. This observation can be the result of a proper psychiatric follow-up, which is in line with previous studies proposing the follow-up as a preventive factor.^35–37^

According to our results, the most frequent comorbidities diagnosed within a same perinatal period are depression and anxiety disorders, which is consistent with previous studies.^4,7,26^ Whereas little is known on perinatal multi-comorbidity,^28^ we identified various combinations of more than two disorders, the most frequent one combining depression, anxiety disorder and obsessive-compulsive disorder. Nevertheless, many other diagnostic combinations have been observed. These findings support evidence for the complexity and the wide range of expressions of psychiatric proneness, thus highlighting the importance of psychiatric screening that goes far beyond depression and anxiety disorders during the whole perinatal period.

According to our clinical trajectories, new psychiatric disorders seem to arise either during pregnancy or postpartum. Indeed, almost all observed clinical trajectories entailed a new diagnosis only during either one of these two perinatal periods despite considering the full range of DSM disorders. This observation may have been tainted by the fact that more than one-third and two-thirds of women in the cohort were under pharmacological treatment during pregnancy and postpartum, respectively, and knowing that the women who discontinue their medication during pregnancy or postpartum are at greater risk.^38,39^

Also, the past psychiatric history could be a precursor of some specific perinatal disorders. A past depressive disorder history was followed by the onset of an anxiety disorder at least twice as often as any other diagnosis during pregnancy and almost three times more often during postpartum. Hence, as outlined in the trajectories, women with an unresolved past psychiatric history that combined with a new perinatal onset diagnosis added to the presence of comorbidity, but beyond the perinatal onset comorbidity described in Table 4. Yet, some women consulted for preventive purposes due to their past psychiatric history and turned out not having any new diagnosis either during pregnancy or postpartum, whereas other women had a first-lifetime psychiatric diagnosis during the postpartum period. This suggests that the postpartum period could be a trigger for psychiatric disorders, but not the pregnancy period. Indeed, all women with a new disorder during pregnancy already had a past psychiatric history, and therefore a psychiatric vulnerability. Hence, our trajectories illustrate the complexity of the psychiatric evolution across the perinatal period and our next study will investigate the risk factors predicting the perinatal onset of severe psychiatric disorders.

It is well documented that an effective treatment during the perinatal period is crucial due to the potential negative consequences of an untreated psychiatric illness on the mother, fetus and infant.^40–43^ We thus described psychotropic medication prescribed in our cohort. The most frequently used ones were Citalopram, Benzodiazepines, Sertraline and Quetiapine, being in line with clinical guidelines where: 1- Sertraline is an often-preferred antidepressant due to its safety and the lower relative dose transferred to the infant during lactation^44–47^; 2- Citalopram is considered to be among reasonable choices of antidepressant^41^; 3- Quetiapine is not associated with an increased risk of congenital malformations^41^; and 4- Benzodiazepines, with the lowest dose and shortest possible half-life, are not an absolute contraindication during the perinatal period.^41^ In our study, whereas 112 women had a past bipolar disorder history and 47 were newly diagnosed during pregnancy, only 5 of them used Lithium during pregnancy. In several studies, Lithium was shown to increase the risk of cardiac malformation in the fetus.^41,48,49^ Alternatives, such as atypical antipsychotics, are therefore preferable, but sometimes the risk of bipolar disorder relapse could outweigh the increased risk of fetal malformation and, thus, the decision to continue Lithium during pregnancy can be well-supported.^41^

*Limitations and strengths*. First, our cohort was composed exclusively of women who required tertiary psychiatric care, so our findings do not generalize to the entire population of women. Moreover, although the information was recorded prospectively in the medical files, our data were collected retrospectively, which did not allow us to determine if a woman had a preexisting undiagnosed disorder that worsened during the perinatal period. For instance, personality, attention-deficit/hyperactivity and premenstrual dysphoric disorders may have been diagnosed during the perinatal period but in a retrospective way, if symptoms were present in the past. The study lacks the trimester of pregnancy, or the exact time frame elapsed after childbirth when the diagnoses were assessed. Also, changes in psychotropics and dosages were not addressed. Future research should specify the precise perinatal moment of a diagnosis and assess the evolution of psychotropic prescriptions across the perinatal period. Despite these limitations, this study benefited from an entire cohort of 964 women and thoroughly exploited the evolution of the full range of disorders clinically diagnosed by a psychiatrist during a medical follow-up, in contrast to previous studies that mostly focused on depression assessed through a screening questionnaire.^10^ Focus was given to the timing of onset of psychiatric disorders in these women (before, during or after pregnancy) in order to better characterize clinical trajectories.

## Conclusion

Our findings highlight the heterogeneity in women's perinatal journey, underscoring the complexity of this period. Access to a specialized perinatal psychiatry clinic, along with the use of psychotropic medications, may offer protective benefits, given the absence of any new disorders during the perinatal period for some women despite a past psychiatric history. However, new mental health issues during pregnancy could be explained by the psychiatric vulnerability associated with a past history. On the other hand, women without prior psychiatric conditions face a different challenge: they could develop a first-ever psychiatric diagnosis during this critical period, making early detection and intervention more difficult. Our results emphasize the need for tailored and proactive care strategies to address the diverse needs of women during the perinatal period.
